# The Prevalence of Thyroid Autoimmunity in Pregnancy and Adverse Neonatal Outcomes at a Secondary Care Hospital in the Middle East

**DOI:** 10.7759/cureus.24814

**Published:** 2022-05-07

**Authors:** Sufia Athar, Stephen F Beer, Zeena Martis, Mohammad I Alloub

**Affiliations:** 1 Obstetrics and Gynecology, Hamad Medical Corporation, Al Wakra, QAT; 2 Endocrinology, Diabetes and Metabolism, Hamad Medical Corporation, Al Wakra, QAT

**Keywords:** thyroid autoimmunity, neonatal outcome, pregnancy, thyroid antibodies, euthyroid, hypothyroid

## Abstract

Background and objective

Among the common endocrinological disorders of pregnancy, thyroid disorders rank second after diabetes. Thyroid autoimmunity is linked to sub-fertility, miscarriages, preterm birth, gestational diabetes, and adverse neurodevelopmental sequelae in children. Peroxidase and thyroglobulin autoantibodies may be associated with enhanced global autoimmune state, which may have adverse effects on the fetal or placental development. It is the main cause of hypothyroidism in reproductive-age women and is associated with poor obstetric outcomes. In Gulf countries, a higher prevalence of thyroid antibodies during pregnancy was reported. However, there is paucity of data in the literature in the Gulf countries in this regard. Our study was conducted to assess the effects of thyroid antibodies on adverse maternal and neonatal outcomes in pregnancy in a multiethnic population of Qatar. The study aimed to assess the prevalence of thyroid antibodies in pregnant hypothyroid women and their impact on adverse fetal outcomes in pregnancy.

Design

A Retrospective study of patients' notes (retrospective chart review) was conducted at a secondary hospital in Qatar. Records of the women who delivered from January 2017 to June 2018 were evaluated. A total of 384 women were included after applying exclusion criteria. Neonatal outcomes were compared in hypothyroid women with (group A1) and without thyroid antibodies (group A2) and were compared with euthyroid women (group B). Statistical analysis was performed using SPSS Statistics version 27.0 (Armonk, NY: IBM Corp).

Results

A total of 7978 women delivered during the study period and the prevalence of hypothyroidism in our sample was 3.47%; 33.33% had thyroid antibodies. Women with more than one miscarriage were 30% (odds ratio {OR}: 2.06, 95% confidence interval {CI}: 1.112-3.811, p<0.05), 21.24% (OR: 1.20, 95% CI: 0.676-2.115, p>0.05), and 17% in group A1, A2, and B, respectively. The incidence of preterm births was 10% (OR: 2.22, 95% CI: 0.760-6.495, p>0.05), 4.23% (OR: 0.94, 95% CI: 0.308-2.876, p>0.05), and 4.5% in groups A1, A2, and B, respectively. Small for gestational age infants were 10% (OR: 3.33, 95% CI: 1.036-10.71, p<0.05), 5.93% (OR: 1.97, 95% CI: 0.640-6.023, p>0.05), and 3% in groups A1, A2, and B, respectively. The study revealed an association between thyroid antibodies and adverse maternal and neonatal outcomes in pregnancy.

Conclusion

Thyroid autoimmunity is associated with poor fetal outcomes. In Gulf countries with higher prevalence of endocrinological disorders (obesity and diabetes), thyroid disorders need attention. As fewer studies were reported from these areas with limited results in literature, this study gives an insight into the prevalence of thyroid disorders, thyroid antibodies, and their association with pregnancy outcomes.

## Introduction

Among the endocrinological disorders in pregnancy, thyroid disorders rank second after diabetes. They are common in the general population with a higher preponderance in females. Interaction between estrogen and thyroid hormones is suggested as the cause of higher incidence in females [1,2]. Thyroid antibodies (TA) are reported in five to 15% of people, with a higher prevalence in elderly females [1,2]. Thyroid peroxidase antibodies (TPO-Ab) were noted in more than 80% of cases, thyroglobulin antibodies (TGA) in >50% of cases, and thyroid-stimulating hormone receptor (TSHR) antibodies in 10% of cases with Hashimoto’s thyroiditis. TSHR antibodies are found in 90% of cases of Graves’ disease. Thyroid peroxidase (TPO), thyroglobulin antibodies (TGA), and TPO antibodies are commonly seen in women with autoimmune thyroid disease (AITD) [3]. In pregnancy, the prevalence of thyroid antibodies is 2% to 17% [3,4]. Nearly 20% of women in the first trimester reveal thyroid antibodies [4]. As the pregnancy advances their levels show a decline and in the third trimester, it reaches a nadir. Their levels rise again in the post-natal period [4].

Thyroid disorders are known to be associated with adverse maternal and neonatal outcomes. The presence of thyroid antibodies worsens these outcomes [4,5]. Even euthyroid women with thyroid antibodies in the first trimester develop sub-clinical hypothyroidism in the second and third trimesters [5]. These thyroid antibodies are correlated with poor fetal outcomes [4,5]. Autoimmune hypothyroidism is associated with miscarriage, preterm birth, intrauterine fetal death (IUFD), and high birth weight in the offspring [4-6].

Multiple mechanisms for adverse outcomes in pregnancy have been described. As the fetal thyroid is functional nearly after 12 weeks of gestation, low maternal thyroxine levels are detrimental to the development of the neurological system of the fetus [6]. In the latter two-thirds of pregnancy, untreated hypothyroidism can lead to cognitive disorders, low intelligence quotient (IQ) levels, activity disorders, and delays in development after birth [6,7]. Thyroid antibodies further potentiate these effects by autoimmune interaction with fetal cells [4,5]. In women with advanced maternal age, the severity increases even more [4,5].

There is a positive correlation between the presence of thyroid antibodies and miscarriage. This effect worsens with increased maternal age [4,5,8]. Women with thyroid antibodies treated with thyroxine have shown lower miscarriage and preterm births in comparison to those not treated [5,6,9,10]. However, some studies have not shown any effect on the miscarriage rate [5]. Better to say there is insufficient data available to make this conclusion. Our study was conducted in a secondary care level hospital in Qatar to assess the effects of thyroid antibodies on pregnancy outcomes.

## Materials and methods

The study is a retrospective chart review, also known as a medical record review. The study was approved by the Institutional Review Board of Medical Research Center, Qatar in 2019 (#MRC-01-18-340). The antenatal patients delivered at a secondary care hospital in Qatar from January 2017 to May 2018 were considered the cohort population. Simple random sampling was used to get the required sample size and to avoid any selection barrier. A total of 7978 women delivered in the study period. Of these, 287 women were diagnosed with hypothyroidism and attended the endocrine clinic. The sample size was 400 patients. Out of these, 200 hypothyroid women were included and 200 women were taken as control by random sampling method. Based on the exclusion criteria 16 hypothyroid pregnant women were excluded. The study population was divided into two main groups; group A which included antenatal patients with hypothyroidism (n=184) and group B (control) which comprised antenatal patients without hypothyroidism (n=200). Group A patients were further divided into those with thyroid antibodies (A1) and those without thyroid antibodies (A2). Post-natal, follow-up was noted at six to eight weeks.

All the pregnant women (18-40 years of age) in the study timeline who had antenatal care in our hospital were included in this study. Patients with recurrent pregnancy loss, overt diabetes, essential hypertension, antenatal care in other hospitals, and incomplete documents/data were excluded. In the control population, euthyroid women with positive thyroid antibodies were excluded. A total of 400 pregnant women were included in the study. After applying exclusion criteria 184 hypothyroid and 200 euthyroid women were part of the study. It was computed based on the effect size (difference in the primary outcome measures between two groups) reported in the literature. Informed consent was not applicable, as the data were retrieved from medical records. Following fetal outcomes; miscarriage, preterm birth (PTB), small for dates infants (SFD), large for date infants (LFD), admissions to neonatal intensive care unit (NICU), and intrauterine fetal death (IUFD) were noted in each group and compared with controls (women without hypothyroidism).

Descriptive statistics were used to summarize and determine the sample characteristics and distribution of parameters related to maternal and neonatal parameters. The normally distributed data and results were reported with mean and standard deviation (SD) with a corresponding 95% confidence interval (CI); the remaining results were reported with median and interquartile range (IQR). Categorical data were summarized using frequencies and percentages. The primary outcome measure was to determine and compare the key fetal outcomes among antenatal women with and without autoimmune hypothyroidism. Comparisons of quantitative data between two groups (antenatal women with and without autoimmune hypothyroidism with those without hypothyroidism) were performed by applying the unpaired t-test or Mann-Whitney U test as appropriate. Associations between two or more qualitative variables were assessed using the chi-square (χ2) test and/or Fisher's exact test as appropriate. The relationship between hypothyroidism and associated factors was estimated by deriving odds ratios (ORs) from the logistic regression model and results were presented and reported with an odds ratio (OR) and associated 95% CI. All p-values presented were two-tailed, and p-values <0.05 were considered statistically significant. All statistical analyses were performed using statistical packages SPSS version 27.0 (Armonk, NY: IBM Corp) and Epi-info (Centers for Disease Control and Prevention: Atlanta, GA) software.

## Results

Demographics of women in the study population

Table 1 shows the demographic characteristics of the hypothyroid women in the study population. Both the groups of women were comparable in all these parameters (p>0.05). Most of the women were in the age range of 26-35 years. In hypothyroid women with and without thyroid antibodies, 80.95% and 84.3% of women were in this range, respectively (p>0.05). Women above 40 years were not included in the study to elude age-related bias. In both groups, nulliparous and multiparous women were in the nearly same proportion. Nulliparous women were 25.4% and 20.66% in each group (p>0.05), while multiparous women were 74.6% and 79.34%, respectively (p>0.05). Women with thyroid antibodies had a higher likelihood of being overweight (33.33%) and obese (34.93%) in comparison to women without thyroid antibodies (overweight: 24.79% and obese: 29.75%). But the results were not statistically significant (p>0.05).

**Table 1:** The demographic characteristics of the hypothyroid women with and without thyroid antibodies in the study population. OR: odds ratio, CI: confidence interval

Demographic		Hypothyroid with thyroid-antibody, n (%)	Hypothyroid without thyroid-antibody, n (%)	OR	95% CI	p-Value
Age (years)	Overall					0.372
	20-25	8 (12.70%)	17 (14.05%)	0.94	0.381-2.324	0.895
	26-30	26 (41.27%)	53 (43.80%)	0.87	0.466-1.614	0.653
	31-35	25 (39.68%)	49 (40.50%)	1.02	0.546-1.909	0.950
	36-40	1 (1.59%)	2 (1.65%)	0.96	0.085-10.793	0.973
Parity	Nulliparous	16 (25.40%)	25 (20.66%)	1.31	0.637- 2.680	0.465
	Multiparous	47 (74.60%)	96 (78.51%)	0.77	0.373- 1.568	0.465
Body mass index (BMI)	Overall					0.267
	Normal weight (BMI <25 kg/m^2^)	20 (31.75%)	55 (45.45%)	0.56	0.294-1.058	0.074
	Overweight (BMI = 25-29.9 kg/m^2^)	21 (33.33%)	30 (24.79%)	1.52	0.779-2.955	0.221
	Obese (BMI >30 kg/m^2^)	22 (34.92%)	36 (29.75%)	1.27	0.663-2.423	0.474

Prevalence of hypothyroidism and thyroid antibodies

The overall prevalence of hypothyroidism in the study population was 3.60% (287 {hypothyroid pregnant women delivered} / 7978 {total women delivered in the study period}). A total of 35% of women had hypothyroidism before pregnancy. And 25.56% of women had the first diagnosis in the first trimester while 22.78% and 16.67% of women were diagnosed in the second and third trimester, respectively. A total of 33.33% of all hypothyroid women had thyroid antibodies. Thyroid peroxidase antibodies (TPO-Ab) were noted in all these cases and thyroglobulin antibodies (TGA) in 4.44% of women. All the women with thyroglobulin antibodies also had TPO-Ab.

Level of thyroid antibodies hormones in the study population

In women with hypothyroidism with thyroid antibodies, the value of TSH ranged from 3.2 to 40.47 mlU/L at the time of diagnosis. The average value of TSH in this group was 8.11 mlU/L. The level of free T4 was in the range of 0.01-15.24 pmol/L, with an average of 11.4 pmol/L. In hypothyroid women without thyroid antibodies, the average level of TSH and free thyroxine (FT4) were 5.55 mlU/L and 11.08 pmol/L, respectively (TSH ranged from 2.62 to 29.56 mlU/L and FT4 ranged from 5.6 to 15.91 pmol/L). In women with thyroid antibodies, higher levels of TSH were noted in comparison to those without thyroid antibodies; levels of FT4 were similar (Table 2).

**Table 2:** Range and average of FT3, FT4, and TSH in the study population. FT3: free triiodothyronine, FT4: free thyroxine, TSH: thyroid-stimulating hormone

Variables	Number of women	Range of FT3	Average FT3 (pg/mL)	Range of FT4	Average FT4 (pmol/L)	Range of TSH	Average TSH mIU/L
Euthyroid women	201	2.87-10.9	4.59	6.11-14.31	10.83	1.11-2.7	1.81
Hypothyroid women without antibody	120	2.48-10.9	4.1	5.6-15.91	11.08	2.62-29.56	5.55
Hypothyroid women with antibody	63	3.08-11.2	4.25	0.01-15.24	11.4	3.2-40.47	8.11

Neonatal outcomes

A comparative analysis was carried out between hypothyroid women with (n=63) or without (n=120) thyroid antibodies and euthyroid women (n=201) were taken as control. Intrauterine fetal deaths (IUFD) were excluded in calculating statistics for preterm birth (PTB), small for dates (SFD) infants, large for dates (LFD) infants, and neonatal intensive care unit (NICU) admissions. Neonatal outcomes were noted in each group and then compared with the control. Statistical analysis was performed.

Neonatal outcomes were compared in hypothyroid women with and without thyroid antibodies and a comparative analysis was done with euthyroid women in the study population. Number of women with previous miscarriages were more frequent in the group with thyroid antibodies (34.04%) as compared to hypothyroid women without thyroid antibodies (25.26%). Hypothyroid women had a higher prevalence of miscarriage as compared to euthyroid women and the results were statistically significant (p<0.05).

Incidence of preterm births was 10%, 4.2%, and 4.5% in hypothyroid women with and without thyroid antibodies and euthyroid women, respectively. A positive correlation was noted between preterm birth and thyroid antibodies, however, the results were not statistically significant (p>0.05). Incidence of small for dates infants were 10%, 5.88%, and 3% in hypothyroid women with and without thyroid antibodies and euthyroid women, respectively. A positive correlation between small for date babies and the presence of thyroid antibodies was noted and the results were statistically significant (OR: 3.592, 95% CI: 1.114-11.588, p<0.05).

The prevalence of large for dates babies in all the three groups was nearly similar (11.73%, 10.92%, and 9%, respectively). A higher incidence of neonatal ICU admissions (with respiratory morbidity) was noted in women with thyroid antibodies (21.67%) and hypothyroid women (15.97%) as compared to euthyroid women (8.5%). Results were statistically significant in both groups of hypothyroid women with (OR: 2.98, 95% CI: 1.351-6.561, p<0.05) and without thyroid antibodies (OR: 2.05, 95% CI: 1.017-4.112, p<0.05). Intrauterine fetal deaths were higher in women with thyroid antibodies (5%, OR: 10, 95% CI: 1.021-97.916, p<0.05) as compared to hypothyroid women without thyroid antibodies and euthyroid women and the results were statistically significant (Table 3).

**Table 3:** Neonatal outcomes in hypothyroid women with and without thyroid antibody in comparison with euthyroid women. Ab: antibody, NICU: neonatal intensive care unit, OR: odds ratio, CI: confidence interval

Variables		Hypothyroid women, n (%)	Euthyroid women, n (%)	OR	95% CI	p-Value
Previous miscarriage	With Ab	16 (34.04%)	27 (15.43%)	2.83	1.364-5.868	0.005
	Without Ab	24 (25.26%)	27 (15.43%)	1.85	0.998-3.439	0.050
Preterm birth	With Ab	6 (10%)	9 (4.5%)	2.36	0.804-6.918	0.118
	Without Ab	5 (4.20%)	9 (4.5%)	0.93	0.304-2.846	0.899
Small for dates	With Ab	6 (10%)	6 (3.00%)	3.59	1.114-11.588	0.032
	Without Ab	7 (5.88%)	6 (3.00%)	2.02	0.663-6.163	0.216
Large for dates	With Ab	7 (11.67%)	18 (9%)	1.34	0.530-3.368	0.540
	Without Ab	13 (10.92%)	18 (9%)	1.24	0.584-2.632	0.575
NICU admission	With Ab	13 (21.67%)	17 (8.5%)	2.98	1.351-6.56	0.007
	Without Ab	19 (15.97%)	17 (8.5%)	2.05	1.017-4.112	0.045
Intrauterine fetal death	With Ab	3 (5%)	1 (0.5%)	10.00	1.021-97.916	0.048
	Without Ab	2 (1.65%)	1 (0.5%)	3.33	0.299-37.156	0.328

Neonatal outcomes were also compared between hypothyroid women with and without thyroid antibodies. Prevalence of preterm labor (10% versus 4.2%), small for dates babies (10% versus 5.88%), NICU admissions (21.67% versus 15.97%), and intrauterine fetal death (5% versus 1.65%) was higher in women with thyroid antibodies as compared to hypothyroid women without thyroid antibodies. However, the results were not statistically significant (p<0.05). No difference was noted in the prevalence of large for date babies in both groups. A higher prevalence of women with previous miscarriages was noted in women with thyroid antibodies as compared to those without thyroid antibodies (34.04% versus 25.26%) (Table 4).

**Table 4:** Neonatal outcomes in hypothyroid women with and without thyroid antibodies. SFD: small for dates, LFD: large for dates, NICU: neonatal intensive care unit, IUFD: intrauterine fetal death

Variables	Hypothyroid women with antibody, n (%)	Hypothyroid women without antibody, n (%)	Odd’s Ratio	95% CI	p-Value
Previous miscarriage	16 (34.04%)	24 (25.26%)	1.53	0.714-3.266	0.275
Preterm birth	6 (10%)	5 (4.20%)	2.51	0.734-8.594	0.142
SFD	6 (10%)	7 (5.88%)	1.78	0.570-5.546	0.322
LFD	7 (11.67%)	13 (10.92%)	0.32	0.406-2.859	0.882
NICU admission	13 (21.67%)	19 (15.97%)	1.46	0.663-3.195	0.349
IUFD	3 (5%)	2 (1.65%)	2.98	0.484-18.287	0.239

## Discussion

Thyroid antibodies are noted in 2-17% of women during pregnancy [10]. But they are also noted in euthyroid women. Euthyroid women with thyroid antibodies autoimmunity are commonly noted to have high TSH levels >4 mU/L during pregnancy. It is also correlated with iodine intake and ethnicity [10]. Levels of TSH in women with thyroid antibodies autoimmunity are also higher than in those without thyroid antibodies [9]. Thyroid antibodies attain peak serum levels in the first trimester and reduce to nearly 60% in the later pregnancy [11,12]. TSH levels in women with thyroid antibodies autoimmunity are accelerated with advancements in the period of gestation. An increase in TSH level from 1.7 mIU/L at 12 weeks to 3.5 mIU/L at term is reported. Hence a four-weekly assessment is recommended during pregnancy [10]. As the placenta is permeable to thyroid antibodies, there is a possibility of its effect on the fetus [13]. Many fetal complications like miscarriages, preterm birth, IUFD, and birth weight disorders are associated with thyroid autoimmunity [5,14].

Rates of miscarriages are higher in women with hypothyroidism [14]. Studies have demonstrated a nearly two-fold increase in thyroid antibodies as compared to women without them [15,16]. The exact mechanism for the increase in miscarriages is not known. However, several theories have been suggested which include high levels of endometrial cytokines, Ab-mediated mild thyroid antibodies hypofunction, non-organ specific autoimmunity, and human chorionic gonadotropin (hCG) receptors cross-immunity by thyroid antibodies in these women [17,18]. Imaizumi et al. have suggested the possibility of fetal resorption which was noted in active immunization in murine [19]. In our study, higher miscarriage rates were noted in women with thyroid antibodies autoimmunity (OR: 2.059, 95% CI: 1.112-3.811, p=0.0215) and the results were statistically significant.

Preterm birth (PTB) is associated with neonatal morbidity, mortality, and long-term effects. It also lays economic impacts on the hospitals and the parents. Despite advancements in obstetrics, preterm birth is still unpredictable. Many studies in the past have correlated PTB with hypothyroidism. The effects of thyroid antibodies on PTB have been evaluated by many authors with variable results. Many authors have reported a higher incidence of PTB in women with thyroid antibodies. Glinoer et al., Ghafoor et al., and Kumru et al. in their studies have demonstrated that women with thyroid antibodies have a higher likelihood of preterm delivery. Their results were statistically significant [20-22].

In a meta-analysis by Negro, Thangaratinam et al., and He et al., an association of preterm births (PTB) with thyroid antibodies was observed. All these studies revealed a positive correlation between PTB with TPO antibodies [23-25]. In our study also a higher incidence of PTB was observed in women with thyroid antibodies. The incidence of PTB was 10% (OR: 2.22, 95% CI: 0.760-6.495, p=0.145) in women with thyroid antibodies, 4.23% (OR: 0.9416, 95% CI: 0.308-2.876, p=0.9159) in women with hypothyroidism without thyroid antibodies, and 4.5% in the control group. The results were not statistically significant. Negro et al. also noted that women with thyroid antibodies who were treated with levothyroxine had a lower incidence of PTB as compared to those who were not treated (7% versus 22.4%, p<0.05) and the result was statistically significant [12]. In this regard, very few studies are available and further studies are required to establish the correlation with PTB.

The effects of hypothyroidism on neonatal birth weight have been studied by many authors. In a few studies, hypothyroidism is correlated with low birth weight (LBW) [26-29], in some with high birth weight (HBW) [30,31], and some have not reported any effect [32]. Karakosta et al. in their study reported that hypothyroid women with thyroid antibodies have three times higher risk for LBW (OR: 3.1, 95% CI: 1.2-8.0) as compared to controls. However, women with normal TSH levels with thyroid antibodies did not affect birth weight [27,28]. Till now, no causal relationship with birth weight has been established. The mechanism behind LFD infants with autoimmunity is suggested to be linked to a high prevalence of obesity and diabetes in women with thyroid antibodies [30,31].

In this study, a higher prevalence of small for dates (SFD) infants was noted in women with thyroid antibodies (10%, OR: 3.33, 95% CI: 1.0368-10.71, p=0.0433) as compared to women without thyroid antibodies (5.93%) and controls (3%). Few studies have noted a correlation between hypothyroidism and large for dates infants. Negro et al. in 2011 noted an insignificant difference in high birth weight neonates in hypothyroid women with and without thyroid antibodies [23]. In our study higher neonatal intensive care (NICU) admissions were noted in women with thyroid antibodies. In some studies, no association was noted [33] and some authors have reported prolonged NICU stay in neonates of hypothyroid women with thyroid antibodies [29,34]. Kiran et al. have noted lower NICU admissions in women with thyroid antibodies [35]. Meena et al. in 2016 have noted poor neonatal outcomes even in euthyroid women [33].

In a study by Janna et al. in 2016, 1025 women with stillbirth were evaluated to assess the utility of thyroid antibody function in this group. Only 2% of women were known to have thyroid antibody disease. But in the rest of the women, nearly 18% of them were diagnosed to have thyroid antibodies abnormality. Out of these, 16.23% had clinical or sub-clinical hypothyroidism [36]. Chen et al. reported a higher incidence of fetal loss in women with overt thyroid antibody dysfunction. With a doubling of TSH levels, a 60% increase in the incidence of child loss was noted in their study [37]. In another study, IUFD was noted in 2.4% of women with thyroid peroxidase antibodies and 3.1% of women with thyroglobulin antibodies. In women without thyroid antibodies, none of them had any fetal loss [35]. In a study by Meena et al., no difference was noted in IUFD in both groups. Most of these studies had a small sample size of women with IUFD [23,33,35,37]. Studies with a larger sample size are recommended in this regard.

Treatment with L-thyroxine has been associated with better neonatal outcomes [38]. Reduced incidence of PTB and miscarriage were observed by Negro et al. after treatment with L-thyroxine [12]. Gartner et al. have reported reduced levels of thyroid antibodies after Selenium supplementation [39]. These women should be monitored every four to six weeks till reference TSH levels are attained and then in every trimester. The dose of therapy should be adjusted with TSH levels. Neonates of these women have a higher incidence of low TSH at birth and thyroid antibodies. Zhang et al. in their study reported that 28% of neonates had a TSH value more than the reference range for age [40]. These neonates need full evaluation and follow-up. Thus, pre-pregnancy control is required to reduce neonatal complications [41].

In the post-natal period, these thyroid antibodies rise again and a study by Chen et al. in 2016 has noted a higher incidence of postpartum thyroiditis in women with thyroid antibodies (42.31% versus 7.14%) in comparison to control [29]. Hence post-natal follow-up at six weeks should be considered mandatory in these women for adjustment of the dose of the therapy. Gulf countries are known to have a high prevalence of population with high body mass index and a high prevalence of endocrinological disorders. Very few studies are published on neonatal outcomes in women with thyroid autoimmunity in this region [42-44]. More studies in this regard in Gulf countries will aid in to conclude.

Strengths and limitation

Our study included a thorough analysis of association between maternal thyroid antibodies and neonatal outcomes. This study has excluded age-related bias as study participants were less than 35 years of age. In advanced maternal age, neonatal outcomes may be poor and the prevalence of thyroid antibodies may be high. As the study was performed on a multi-ethnic population, ethnicity-related bias was excluded. In Gulf countries with higher prevalence of endocrinological disorders e.g., obesity and diabetes, thyroid disorders need attention and fewer studies were reported with limited results. This study gives an insight into the prevalence of thyroid disorders, thyroid antibodies, and its association with pregnancy outcomes. The limitation of this study is that it was retrospective and of a reasonable sample size. Future studies with a larger sample size will aid in clearer recommendations.

## Conclusions

It is well established that hypothyroidism in pregnancy is associated with maternal and neonatal complications. These complications may be worsened in women with thyroid antibodies. Attaining normal TSH levels during pregnancy through adequate and timely management could possibly reduce these complications. Hence a combined strategy of maternal and fetal surveillance should be implemented on these women. A multidisciplinary approach involving an obstetrician, physician endocrinologist, and neonatologist should be planned for this cohort of women. This should also include a post-natal follow-up. Close monitoring of TSH levels in each trimester and appropriate adjustments in the therapy is the key to preventing neonatal complications. We provide further evidence that thyroid autoimmunity in our heterogeneous population in the Middle East, is associated with adverse outcomes in both early and late pregnancy, and suggest that further research into both the prevalence of these complications and the effect of thyroxine treatment is undertaken. In euthyroid women with thyroid antibodies in pregnancy, the initiation of the therapy is not yet recommended because of insufficient evidence to reduce miscarriage and PTB. Future studies in this regard may draw some clear consensus.
